# Phenolic Fingerprint, Bioactivity and Nanoformulation of *Prunus spinosa* L. Fruit Extract for Skin Delivery

**DOI:** 10.3390/pharmaceutics15041063

**Published:** 2023-03-25

**Authors:** Maria De Luca, Carlo Ignazio Giovanni Tuberoso, Ramon Pons, María Teresa García, María del Carmen Morán, Giulio Ferino, Antonio Vassallo, Giuseppe Martelli, Carla Caddeo

**Affiliations:** 1 Department of Science, University of Basilicata, Viale dell’Ateneo Lucano 10, 85100 Potenza, Italy; 2 KAMABIO Srl, Via Al Boschetto 4/B, 39100 Bolzano, Italy; 3 Department of Life and Environmental Sciences, University of Cagliari, SS 554–bivio per Sestu, Monserrato, 09042 Cagliari, Italy; 4 Department of Surfactants and Nanobiotechnology, Institute for Advanced Chemistry of Catalonia (IQAC-CSIC), c/Jordi Girona, 18-26, 08034 Barcelona, Spain; 5 Department of Biochemistry and Physiology, Physiology Section, Faculty of Pharmacy and Food Science, University of Barcelona, Avda. Joan XXIII 27–31, 08028 Barcelona, Spain; 6 Institute of Nanoscience and Nanotechnology-IN2UB, University of Barcelona, Avda. Diagonal, 645, 08028 Barcelona, Spain; 7 CeSAR, University of Cagliari, SS 554–Bivio per Sestu, Monserrato, 09042 Cagliari, Italy; 8 Spinoff TNcKILLERS s.r.l., Viale dell’Ateneo Lucano 10, 85100 Potenza, Italy

**Keywords:** *Prunus spinosa* berry extract, phenolic composition, antioxidant, antibacterial, phospholipid vesicles, biocompatibility, skin delivery

## Abstract

The nanoformulation of plant extracts in phospholipid vesicles is a promising strategy to exploit the biological properties of natural bioactive substances and overcome drawbacks such as poor aqueous solubility, chemical instability, low skin permeation and retention time, which strongly limit their topical application. In this study, *Prunus spinosa* berries were used for the preparation of a hydro-ethanolic extract, which showed antioxidant and antibacterial properties owing to the presence of phenolic compounds. Two types of phospholipid vesicles were developed to improve the applicability as topical formulations. Liposomes and Penetration Enhancer-containing Vesicles were characterized for mean diameter, polydispersity, surface charge, shape, lamellarity, and entrapment efficiency. Additionally, their safety was assayed with different cell models, including erythrocytes and representative skin cell lines.

## 1. Introduction

*Prunus spinosa* L. is a perennial deciduous plant growing as a thorny bush or small tree in uncultivated areas of different countries, among them the Mediterranean. Also known as blackthorn or sloe, it belongs to the Rosaceae family. Its fruit is a small, spherical, bluish-black drupe with a yellow-greenish pulp and a characteristic astringent flavor, typically processed into jams, jellies, juices, tea, and alcoholic beverages; it has been recently studied as natural food colorant [1,2,3,4,5].

In European tradition, *P. spinosa* is known as a medicinal plant for its diuretic, laxative, antispasmodic, antimicrobial, anti-inflammatory, and antioxidant properties [4,6,7]. *P. spinosa* fruits have been recommended in the treatment of various inflammation-related disorders of the gastrointestinal, urinary, and respiratory tracts, and for oral and pharyngeal mucosa inflammation [1,8,9]. The fruits have also been used to treat metabolic diseases such as diabetes and obesity, and circulatory system disorders [1,10]. More recently, many studies have also found beneficial effects on the wound healing process [6,11], cytotoxic activity on some cancer cell lines [2,9,12,13], and selective growth inhibition of some potentially pathogenic bacteria strains [4]. All these properties can be ascribed to the high content of phenolic acids and flavonoids, including anthocyanins, flavonols, and flavones, found in *P. spinosa* berries [2,4,9,14].

Generally, the main obstacles to the use of phytochemicals in therapy are their chemical instability, vulnerability to environmental conditions, and poor aqueous solubility, alongside the contrast with the bio-pharmaceutical requirements for dermal delivery (e.g., not too high molecular weight nor too high miscibility in the aqueous phase or too low lipid solubility). The incorporation of biologically active compounds in lipid-based nanocarriers is a successful approach to tackling these problems [15,16,17]. In this study, a hydro-ethanolic extract from *P. spinosa* berries was prepared and characterized by identifying compounds commonly known for their biological importance. The antioxidant and antibacterial activities of the extract were verified. Thereafter, two types of phospholipid vesicles were prepared with the aim to improve extract bioavailability in a potential topical application. The nanoformulations were characterized for the most important parameters that guarantee the performance of the nanocarriers, such as mean size and size distribution, charge, shape, lamellarity, and entrapment efficiency. In addition, their safety was assayed in model cells, such as erythrocytes, keratinocytes, and fibroblasts.

## 2. Materials and Methods

### 2.1. Materials

Ethanol absolute was purchased from VWR (Milano, Italy); methanol and 85% *w*/*w* phosphoric acid were purchased from Sigma-Aldrich (Steinheim, Germany). LC-MS grade acetonitrile, formic acid, and water were purchased from Merck (Darmstadt, Germany). Standards of cyanidin-3-*O*-glucoside, cyanidin-3-*O*-rutinoside, peonidin-3-*O*-glucoside, peonidin-3-*O*-rutinoside, quercetin-3-*O*-rutinoside, quercetin-3-*O*-glucoside, quercetin-3-*O*-rhamnoside, *p*-coumaric acid, ferulic acid, vanillic acid, 3-*O*-caffeoylquinic acid (neochlorogenic acid), and 5-*O*-caffeoylquinic acid (chlorogenic acid) were purchased from Extrasynthese (Genay, France) and TransMIT (Giessen, Germany).

Soy lecithin (a mixture of polar phospholipids and glycolipids) and propylene glycol (PG) were purchased from Galeno Srl (Comeana, Prato, Italy).

### 2.2. Extract Preparation

The extraction process was performed through the conventional method of maceration in a solvent. The berries of *Prunus spinosa* L. were collected fully ripened from the Marmo Platano area (Basilicata, Italy) in December 2021. The fruits were cleaned and ground in a mortar with 70:30 *v*/*v* ethanol:water (1:2 *w*/*v* fruits:solvent ratio: 50 g in 100 mL), sonicated (Ultrasonic Processor, UH-500B, Altivole, Treviso, Italy) for 30 min, and macerated for 24 h, at room temperature. After filtration through filter paper, another aliquot of ethanol-water was poured on the exhausted fruits and the procedure was repeated twice. The filtered extractive solutions were joined and subjected to vacuum distillation at 37 °C (Heidolph Laborota 4001 efficient, Schwabach, Germany). The obtained extract (8 g) was stored at −20 °C until use.

### 2.3. LC-High-Resolution MS Analysis (HRMS)

The qualitative investigation of *P. spinosa* fruit extract was performed by an ion mobility QTOF LC-MS system using a 1290 Infinity II UPLC equipped with an autosampler (G7167B), a quat pump (G7120A), a column comp (G7116B) and 6560 IM-QTOF (Agilent Technologies Inc., Palo Alto, CA, USA). Overall instrument performances were tested before analysis using an Agilent tuning solution mix (G1969-85000), and during the analysis, two reference masses at *m*/*z* 112.9855 and *m*/*z* 966.0007 were continuously infused into the system for constant mass correction. The electrospray ionization (ESI) source in negative ion mode was used to perform the analyses and the optimized source parameters were the following: drying gas at 300 °C with a 5 L/min flow, sheath gas at 250 °C with a 12 L/min flow, nebulizer at 45 psi, capillary voltage of 3500 V with a nozzle voltage of 500 V. The automatic acquisition MS/MS experiments were carried out by applying a formula to determine the collision energy by linear interpolation, calculated according to the following equation: collision energy = [slope (5) × *m*/*z* of precursor mass]/100 + Offset (2). The MS spectra were acquired by full range acquisition in a 40–1300 *m*/*z* range.

Chromatographic separation was performed on a Kinetex EVO C18 column (150 × 2.1 mm, 1.7 µm, 100 Å; Phenomenex, Castel Maggiore, Bologna, Italy) maintained at 55 ± 1 °C. The mobile phase was a combination of solvent A (0.1% formic acid) and solvent B (acetonitrile + 0.1% formic acid) at an 0.3 mL/min flow rate. The gradient elution was the following: 0–20 min (99–80% A), 20–35 min (80–70% A), 35–40 min (70–1% A), 40–45 min (1–1% A), 45–46 min (1–99% A) and 46–50 min (99–99% A). The injection volume was 4 µL.

Data acquisition and processing were performed using the MassHunter Workstation Acquisition software v. B.09.00. (Agilent Technologies, Santa Clara, CA, USA). Analysis of ESI/QTOF MS data was performed using the molecular feature extraction algorithm of the MassHunter Workstation Qualitative Analysis software v. 10.0 (Agilent Technologies). The tentative identification and LC-MS/MS analysis of the metabolites were carried out using the MassHunter METLIN metabolite PCDL database v. B.08.00 (Agilent Technologies) and Sirius^®^ software v. 4.7.4 to predict fragmentation and molecular formulae [18,19].

### 2.4. HPLC-DAD Analysis

The quantitative analysis of targeted phenolic compounds was performed using a previously described HPLC-DAD method [20] using a 1260 Infinity II HPLC system equipped with a pump (G7111A), an autosampler (G7129A), a thermostated column compartment (G7116A; 30 ± 1 °C), and a photodiode array detector (G4212B) (Agilent Technologies, Cernusco sul Naviglio, Milan, Italy). The chromatographic separation was conducted on a Kinetex EVO C18 column (150 × 4.60 mm, 2.6 μm, Phenomenex) using a mobile phase of 0.22 M phosphoric acid (solvent A) and acetonitrile (solvent B) at an 0.8 mL/min flow rate. Solvent A was decreased from 100% to 80% in 20 min, to 70% in 35 min, to 0% in 45 min, and then kept stable up to 50 min; the gradient was brought back to 100% solvent A and kept stable for 5 min before the next injection. The injection volume was 10 μL. The chromatograms and spectra were processed using an OpenLab CDS software v. 2.5 (Agilent Technologies), and phenolic compounds were detected and quantified based on absorption at characteristic wavelengths as anthocyanins (520 nm), flavonols (360 nm), hydroxycinnamic acids (313 nm), and hydroxybenzoic acids (280 nm). Stock standard solutions and working standard solutions were prepared in methanol and in 0.22 M phosphoric acid, respectively. The calibration curves were built by correlating the peak area with the concentration by the least squares method, with r^2^ > 0.999 in a 2–500 mg/L range for all the standards. For the analysis, the extract was dissolved with an 80:20 *v*/*v* MeOH:H_2_O mixture (1:50 *w*/*v* extract:solvent ratio) and diluted 1:1 *v*/*v* with 0.22 M phosphoric acid_._ The nanoformulations were injected after dilution (1:100) with a 50:50 *v*/*v* MeOH:H_2_O mixture. The solutions were filtered with a 0.22 μm CA syringe filter before injection.

### 2.5. DPPH Assay

The antioxidant power of the *P. spinosa* fruit extract was examined with the DPPH test by monitoring the reduction reaction of the DPPH radical (1,1-diphenyl-2-picrylhydrazyl): a DPPH solution is discolored as a function of the antioxidant power of the sample, leading to a decrease in absorbance (A) at 517 nm.

A 20 mg/mL solution of *P. spinosa* fruit extract in a 70:30 (*v*/*v*) ethanol:water mixture was prepared. Then, 5 µL of the solution was mixed with a 25 µM DPPH methanolic solution (2 mL) and stored in the dark for 30 min, at room temperature. The discoloration of the DPPH was monitored spectrophotometrically. The antioxidant activity (AA) of the *P. spinosa* fruit extract was calculated according to the following Formula (1):

(1) $$\mathrm{AA}\;\left(\%\right)=\left(\frac{A_{DPPH}-A_{sample}}{A_{DPPH}}\right)\times 100$$

The results were also expressed as Trolox Equivalents (µg TE/mL solution) calculated by means of a calibration curve (Trolox concentration range: 0–1000 µg/mL).

### 2.6. MIC and MBC Determination

The antibacterial activity of the *P. spinosa* fruit extract was assayed by using eight bacterial strains typical of surfaces and skin. The selected microorganisms were *Bacillus subtilis* (ATCC 6633; American Type Culture Collection, Manassas, VA, USA), *Staphylococcus epidermidis* (ATCC 12228), *Staphylococcus aureus* (ATCC 6538), *Listeria monocytogenes* (ATCC 15313), *Enterococcus faecalis* (ATCC 29212), *Escherichia coli* (ATCC 25922), *Acinetobacter baumannii* (ATCC 19606), and *Klebsiella aerogenes* (ATCC 13048). The antibacterial activity was assessed in vitro by the determination of the minimum inhibitory concentration (MIC) using the broth microdilution method [21,22]. Muller Hinton broth (21 g of powder in 1 L of distilled water with a final pH of 7.3, as per manufacturer’s instructions; Oxoid Ltd., Basingstoke, UK) was used as the bacterial growth and dilution medium. Several dilutions of the extract in the broth were made to have a concentration range of 4–16,000 μg/mL in the microtiter plates. Then, 40 μL of the broth culture of each bacterial strain was added to have a final density of approx. 10^6^ colony forming units/mL. Broth with and without bacterial inoculum served as growth and sterility controls, respectively. The MIC value was calculated as the lowest concentration of extract that inhibits the visible growth of the microorganism after 24 h of incubation at 37 °C, also confirmed with resazurin, an indicator of cellular metabolic ability [23]. The dilutions were plated on agar Muller Hinton plates and incubated for 24 h at 37 °C for the determination of the minimum bactericidal concentration (MBC), which is the lowest concentration that kills 99.9% of the inoculum.

### 2.7. Phospholipid Vesicle Preparation and Characterization

Liposomes (lip) and Penetration Enhancer-containing Vesicles (PEVs) were developed. The liposomes were prepared by dispersing soy lecithin and *P. spinosa* fruit extract in ultrapure water (MilliQ RG system, Millipore, Bedford, MA, USA) (Table 1) and sonicating with a Soniprep 150 plus disintegrator (MSE Crowley, London, UK; 10 cycles of 5 s on/2 s off + 5 cycles 2 s on/2 s off; 13 µm probe amplitude).

For the preparation of the PEVs, the penetration enhancer propylene glycol (PG) was included in the formulation. PG-PEVs were prepared according to the protocol used for liposomes’ preparation, but with the addition of 10% *v*/*v* PG.

Empty vesicles were prepared according to the procedure used for extract-loaded vesicles, but without the extract (Table 1), to allow proper comparisons.

The formation and the morphology of the vesicles were assessed by cryogenic-transmission electron microscopy (cryo-TEM). The samples were observed with a JEM-2011 TEM (JEOL USA Inc., Peabody, MA, USA). Then, 4 μL of vesicle dispersion was applied on a holey carbon grid, blotted and plunge-frozen into liquid ethane (−180 °C) using a Leica EM GP cryo-preparation chamber (Leica Microsystems Inc., Deerfield, IL, USA). The sample was embedded in a thin layer of vitreous ice to prevent radiation damage and to preserve the vesicle structure. The analysis was carried out at an accelerating voltage of 200 kV.

The average diameter, polydispersity index, and zeta potential of the vesicles were measured by dynamic and electrophoretic light-scattering techniques (DLS and ELS, respectively) using a Zetasizer nano-ZS (Malvern Panalytical, Worcestershire, UK). The vesicle dispersions were diluted (1:100) with water and analyzed at 25 °C.

The *P. spinosa* fruit extract components non-incorporated in the vesicles were removed via dialysis. Each *P. spinosa* formulation (1 mL) was loaded into Spectra/Por^®^ tubing (12,000–14,000 Da MWCO; Spectrum, Breda, The Netherlands) and dialyzed against water (2 L), under stirring, for 2 h. Non-dialyzed and dialyzed vesicle dispersions were diluted (1:100) with a 50:50 *v*/*v* methanol:water mixture and analyzed by HPLC-DAD to quantify phenolic compounds, according to the procedure described in Section 2.4. To calculate the entrapment efficiency (EE) of the nanoformulations, the following formula was applied (2):

(2) $$\mathrm{EE}=\left(\frac{\mathrm{quantity}\;\mathrm{of}\;\mathrm{phenolic}\;\mathrm{compound}\;\mathrm{in}\;\mathrm{dialyzed}\;\mathrm{vesicles}}{\mathrm{quantity}\;\mathrm{of}\;\mathrm{phenolic}\;\mathrm{compound}\;\mathrm{in}\;\mathrm{non}-\mathrm{dialyzed}\;\mathrm{vesicles}}\right)\times 100$$

### 2.8. Small-Angle X-ray Scattering

A deeper structural characterization of the vesicles was performed by Small-Angle X-ray Scattering (SAXS) analyses. Measurements were carried out using an S3-MICRO (Hecus X-ray Systems GmbH, Graz, Austria) coupled to a GENIX-Fox 3D X-ray source (Xenocs, Grenoble, France) working at 50 kV and 1 mA and providing a detector-focused X-ray beam with a λ = 0.1542 nm Cu K_α_ line with more than 97% purity and less than 0.3% K_β_. Transmitted scattering was detected by using a PSD-50 detector (Hecus X-ray Systems GmbH). Temperature was controlled by a TCCS-3 Peltier (Hecus X-ray Systems GmbH), and diffraction patterns were recorded at 25 °C. The vesicle dispersions were loaded into a flow-through glass capillary (1 mm diameter and 10 μm wall thickness). The scattering curves are shown as a function of the scattering vector modulus *q*, according to the following Formula (3):

(3) $$q=\left(\frac{4\pi }{\lambda }\right)\times \sin\left(\frac{\theta }{2}\right)$$

where *θ* is the scattering angle and λ the wavelength. The *q* values ranged from 0.01 to 0.6 Å^−1^. The scattering vector was calibrated by measuring the standard silver behenate. The scattering curves were recorded every 20 min up to 2 h, the appropriate background was subtracted, and the results were analyzed using a home-made fitting procedure based on a Gaussian description of the bilayers and using a Levenberg–Marquardt minimization scheme [24,25,26,27,28] that includes the pertinent smearing corrections.

### 2.9. Hemolytic Activity

Erythrocytes were isolated from rabbit blood samples, washed three times in Phosphate Buffered Saline (PBS) at pH 7.4, and resuspended in PBS at a cell density of approximately 10^9^ cells/mL.

The hemolytic activity of the *P. spinosa* samples was evaluated by using a procedure described in the literature [29]. The samples under investigation were dispersed in a total volume of 1 mL with PBS and 25 μL of the erythrocyte suspension. The test was performed using 1000 and 2000 µg/mL of the extract solution (ethanol:water 70:30 *v*/*v*) or the extract nanoformulations, and the appropriate controls (100% hemolysis: erythrocytes in Milli-Q water; 0% hemolysis: erythrocytes in PBS buffer). The samples were incubated for 10 min at room temperature, under stirring, and then centrifuged (5 min at 10,000 rpm). The hemolysis (%) was calculated by comparing the absorbance at 575 nm of the supernatant of the *P. spinosa* samples with that of the controls.

Rabbit blood samples were supplied by the Animal Facility of the Research and Development Center (CID)—Spanish National Research Council (CID-CSIC, Barcelona, Spain). The blood samples were obtained in strict compliance with the bioethical principles established by the Spanish legislation. This study was approved by the Animal Experimentation Ethics Committee of the Research and Development Center (CEEA-CID, CSIC).

### 2.10. Skin Cells Viability

Cell viability was assessed by the MTT assay. The latter relies on the mitochondrial ability of live cells to convert yellow 2,5-Diphenyl-3-(4,5-dimethyl-2-thiazolyl) tetrazolium bromide (MTT) into insoluble purple formazan, detectable via spectrophotometry.

Murine Swiss albino fibroblasts (3T3), immortal human keratinocytes (HaCaT), and squamous carcinoma cells (A431) were obtained from Celltec UB (Barcelona, Spain). The cells were cultured in Dulbecco’s Modified Eagle’s Medium with 4.5 g/L glucose (DMEM) plus 10% (*v*/*v*) fetal bovine serum (FBS), 2 mM *L*-glutamine, 100 U/mL penicillin, and 100 μg/mL streptomycin, at 37 °C and 5% CO_2_. When approximately 80% confluence was reached, the cells were harvested using trypsin-EDTA. All these reagents were provided by Lonza (Verviers, Belgium).

The cells were seeded at defined densities (3T3 and HaCaT cells at 1 × 10^5^ cells/mL, A431 cells at 5 × 10^4^ cells/mL) into 96-well plates. After 24 h, the spent medium was removed, and the cells were incubated for another 24 h with the samples under investigation (i.e., *P. spinosa* fruit extract in solution (ethanol:water 70:30 *v*/*v*) or in liposomes or in PG-PEVs), previously diluted in the culture medium to achieve the required concentrations (1–200 μg/mL). After the incubation time, the medium was removed and 100 μL of MTT (5 mg/mL in PBS), diluted (1:10) in DMEM without phenol red and FBS, was added to the cells. After 3 h, the MTT was removed and 100 μL of dimethylsulfoxide was added to each well to dissolve the purple formazan crystals. The plates were shaken for 5 min at room temperature to allow the total dissolution. The absorbance of the solutions was recorded at 550 nm using a Bio-Rad 550 microplate reader (Hercules, CA, USA). The effect of the *P. spinosa* samples was expressed as the percentage of MTT reduction by viable cells against the untreated control cells (cells with medium only; 100% viability).

### 2.11. Statistical Analysis

Results are reported as means ± standard deviation (SD). Student’s *t*-test was used to determine the significant difference between groups. For cell experiments, results are reported as means ± standard error (SE). One-way analysis of variance (ANOVA) was used to determine the significant difference between data sets, following the Scheffé post-hoc test for multiple comparisons. *p*-values below 0.05 were considered statistically significant.

## 3. Results

### 3.1. Quali-Quantitative Determination of Phenolic Compounds in P. spinosa Fruit Extract

The *P. spinosa* fruit extract was qualitatively analyzed by (HR) LC-ESI-QTOF MS/MS in negative ion mode, and targeted phenolic compounds were quantified by HPLC-DAD analysis.

**Figure 1:**
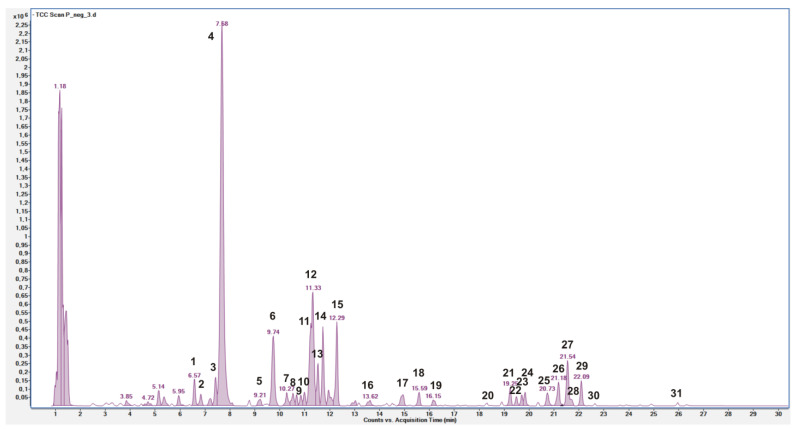
LC QTOF MS Total Compound Chromatogram of *P. spinosa* fruit extract acquired in negative ion mode. Peaks identification is given in Table 2.

The LC-MS profile showed the presence of a large number of compounds corresponding to the deprotonated molecular ions of phenolic derivatives, mainly hydroxycinnamic acid and flavonoid derivatives (Figure 1). Twenty-nine compounds were identified by comparing the *m*/*z* values in the total compound chromatogram (TCC) profile with those described in the literature, and by comparing experimental MS/MS spectra with fragmentation patterns reported in the literature or with the fragmentation patterns and spectra reported in a public repository of mass spectral data [19,30]. Two compounds remain unknown. Table 2 reports the identified compounds with their retention times, the chemical formula derived by mass measurement (experimental result), MS/MS results, mass error (Δ ppm), the references used for identification, and the identification confidence levels (Level 1: reference standard match; Level 2: matched to literature data or databases; Level 3: most likely structure match; Level 4: unknown feature of interest) [31].

Compounds 2, 4, 6, 8, 10–12 and 16–19 were identified as hydroxycinnamic derivatives. Peaks 2, 4 and 12 were identified as caffeoylquinic acid isomers, due to the [M-H]^−^ at *m*/*z* 353.0885 and a fragment at *m*/*z* 191.0532 (loss of a quinic acid unit) [33,34]. By comparison with pure standards, peaks 4 and 12 were attributed to 3-*O*-caffeoylquinic acid (neochlorogenic acid) and 5-*O*-caffeoylquinic acid (chlorogenic acid), respectively. According to Magiera et al. [1], peak 2 was tentatively attributed to *cis*-3-*O*-caffeoylquinic acid. Peak 8, with [M-H]^−^ *m*/*z* 341.0878 and fragments at *m*/*z* 179.0346 and 135.0448, was tentatively attributed to a caffeic acid hexoside [1], and peak 10, with [M-H]^−^ *m*/*z* 515.1399 and fragments at *m*/*z* 341.0853, 191.0554, and 179.0339, was tentatively attributed to a caffeoylquinic acid hexoside [18]. Peaks 5, 6 and 16 were tentatively attributed to coumaroylquinic acids due to the [M-H]^−^ at *m*/*z* 337.0940, and more precisely peak 6 to 3-*p*-coumaroylquinic acid for the characterizing fragment at *m*/*z* 163.0403 [33,34], and peak 16 to 4-*p*-coumaroylquinic acid for the characterizing fragment at *m*/*z* 173.0461 [33,34]. Peak 11, with [M-H]^−^ *m*/*z* 367.1043 and a fragment at *m*/*z* 193.0516, was tentatively attributed to 3-*O*-feruloylquinic acid [33,34]. Compounds 17 and 18, with [M-H]^−^ *m*/*z* 335.0773 corresponding to C_16_H_16_O_8_, and MS/MS product ions at *m*/*z* 179.0352, 135.0448, and 161.0247, were tentatively attributed to caffeoylshikimate isomers [1,37]. Peak 19, with [M-H]^−^ *m*/*z* 381.1186 and a fragment at *m*/*z* 161.0246, was tentatively attributed to ethyl caffeoylquinate (ethyl chlorogenate) [38], a compound previously not detected in *P. spinosa* fruits.

Table 3 reports the quantitative data of targeted phenolic compounds detected in *P. spinosa* fruit extract. Moreover, 3-*O*-caffeoylquinic (neochlorogenic) acid was the most represented hydroxycinnamic derivative and phenolic compound as well (2.38 ± 0.02 mg/g), followed by other quinic acid derivatives, such as 3-*p*-coumaroylquinic acid (0.13 ± 0.00 mg/g), 5-*O*-caffeoylquinic (chlorogenic) acid (0.13 ± 0.00 mg/g), and 3-*O*-feruoilquinic acid (0.07 ± 0.00 mg/g).

The other three compounds (1, 3 and 13) were tentatively attributed to benzyl and benzoic derivatives. Compound 1, with [M-H]^−^ *m*/*z* 329.0877 and a fragment at *m*/*z* 167.0349, was tentatively attributed to vanillic acid-*O*-glucopyranoside [1,32]. This compound was the only hydroxybenzoic acid dosed by HPLC-DAD, and its amount was 0.12 ± 0.00 mg/g (Table 3). Compound 3, with [M-H]^−^ *m*/*z* 313.0932 and fragments at *m*/*z* 101.0245 and 59.0140, was tentatively attributed to a phenolic glycoside derivative [18]. Compound 13, with [M-H]^−^ *m*/*z* 461.1302 and a fragment at *m*/*z* 121.0295, was tentatively attributed to a hydroxybenzoyl-hexosyl-hexoside compound [35] and it was not previously detected in *P. spinosa* fruit. Compounds 14 and 15, with [M-H]^−^ *m*/*z* 447.1508 corresponding to C_19_H_28_O_12_ and two characterizing fragments at *m*/*z* 71.0141 and 101.0242, were tentatively attributed to isomeric forms of barlerin (8-*O*-acetylshanzhiside methyl ester) [36], an iridoid already detected in in the genus *Prunus* [39].

Compounds 20–31 were identified as flavonoid derivatives, more precisely glycoside derivatives of quercetin, by the diagnostic [M-H]^−^ ions shown in the (HR) ESI-MS and MS/MS analysis in negative ion mode, compared with literature data. By comparison with pure standards, compounds 21, 22 and 29 were attributed to quercetin-3-*O*-rutinoside (rutin), quercetin-3-*O*-glucoside and quercetin-3-*O*-rhamnoside (quercitrin), respectively. These compounds were previously reported in *P. spinosa* fruits [1,33,34]. Interestingly, another two compounds (20 and 28) showed the same [M-H]^−^ at *m*/*z* 609 corresponding to C_27_H_30_O_16_ and the two characterizing fragments at *m*/*z* 300 and 301 of quercetin-3-*O*-rutinoside. Thus, compounds 20 and 28 were tentatively attributed to quercetin disaccharides derivatives containing rhamnose and a hexose [1,33,34]. Compound 24 showed a fragmentation pattern very similar to that of quercetin-3-*O*-glucoside: a comparison with quercetin-3-*O*-galactoside retention time excluded the hypothesis that it could be this compound, and it was attributed to a quercetin hexoside. Compound 23, with [M-H]^−^ *m*/*z* 595.1303 and a fragment at *m*/*z* 415.0632, was tentatively attributed to a quercetin disaccharides derivative containing a pentoxide (probably xylose or arabinose) and a hexose (probably glucose or galactose), as previously reported [1,33,34]. Compounds 25–27, with the same [M-H]^−^ at *m*/*z* 433.0773 corresponding to C_20_H_18_O_11_, were attributed to different quercetin pentosides, and quercetin 3-*O*-arabinoside (guaijaverin or reinutrin) was previously detected in *P. spinosa* fruits [1,33,34]. Finally, compounds 30 and 31, with [M-H]^−^ at *m*/*z* 505.0992 and [M-H]^−^ at *m*/*z* 651.1561 corresponding to C_23_H_22_O_13_ and C_29_H_32_O_17_, respectively, were attributed to acetyl derivatives of quercetin hexoside (30) and quercetin hexosyl-rhamnoside (31). These two compounds were already identified by Guimarães et al. [34].

Quercetin-3-*O*-rutinoside (21) was the most abundant flavonol (0.74 ± 0.00, mg/g), followed by quercetin-3-*O*-rhamnoside and quercetin-3-*O*-glucoside (Table 3). The sum of quercetin pentosides (0.49 ± 0.03 mg/g) accounted for 37% of total flavanols, with compound 27 contributing for 60% of this amount. These findings are in accordance with literature data, where high amounts of quercetin-3-*O*-rutinoside and quercetin pentosides were detected in *P. spinosa* fruit extracts [34].

HPLC-DAD analysis showed the presence of four anthocyanins that were not detected by (HR) LC-ESI-QTOF MS/MS in negative mode. The four anthocyanins, identified by comparison with literature data [33,34] and pure standards, were cyanidin-3-*O*-glucoside, cyanidin-3-*O*-rutinoside, peonidin-3-*O*-glucoside, and peonidin-3-*O*-rutinoside, respectively. The most abundant anthocyanin was cyanidin-3-*O*-rutinoside (0.74 ± 0.03 mg/g), followed by peonidin-3-*O*-rutinoside and cyanidin-3-*O*-glucoside, with very similar amounts (0.44 ± 0.01 and 0.43 ± 0.01 mg/g, respectively), and peonidin-3-*O*-glucoside (0.11 ± 0.00 mg/g) (Table 3).

### 3.2. Antioxidant Activity

The antioxidant activity of the *P. spinosa* fruit extract was estimated as a function of its radical scavenging activity. The extract solution scavenged the DPPH radical markedly: 85% ± 1.7 corresponding to 983 μg/mL of Trolox equivalents.

### 3.3. Antibacterial Activity

The antibacterial activity of the *P. spinosa* fruit extract was evaluated against a panel of clinically relevant Gram-positive and Gram-negative bacteria.

The extract showed antibacterial activity against *S. aureus* and *S. epidermidis* (Table 4). More precisely, MIC and MBC were 16 mg/mL for both strains. Based also on these results, we prepared the nanoformulations with an extract concentration of 20 mg/mL, which is above the MIC and MBC values, so it is reasonable to expect that they would have antibacterial activity.

### 3.4. Phospholipid Vesicle Characterization

*P. spinosa* nanoformulations were characterized for mean diameter, polydispersity index, and zeta potential through DLS and ELS measurements (Table 5). The *P. spinosa* liposomes’ mean diameter was less than 100 nm, significantly larger than the empty liposomes, although both appeared monodispersed (polydispersity index <0.02) and negatively charged. The addition of PG to the formulation produced vesicles significantly smaller in diameter than liposomes (86 vs. 94 nm), but with similar values of polydispersity and surface charge. The extract’s loading significantly increased the mean diameter of the PEVs as well (86 vs. 59 nm), keeping unaltered polydispersity index and zeta potential values.

Nine phenolic compounds identified in *P. spinosa* fruit extract were quantified in the non-dialyzed and dialyzed nanoformulations and used to determine the entrapment efficiency (Table 6). Liposomes displayed entrapment efficiencies higher than those of PG-PEVs, with values >96% and >82% for anthocyanins and flavonols, respectively.

**Figure 2:**
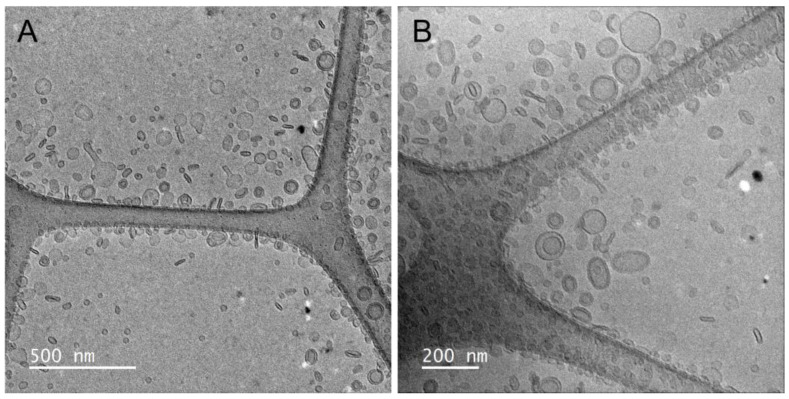
Cryo-TEM images of *P. spinosa* PG-PEVs. Two magnifications are shown: 15,000× (**A**) and 20,000× (**B**).

The morphology of the vesicles was determined by cryo-TEM observations. Representative images of PG-PEVs are shown in Figure 2. Both spherical and elongated unilamellar vesicles were evident, with size below 100 nm, which aligns with DLS data.

**Figure 3:**
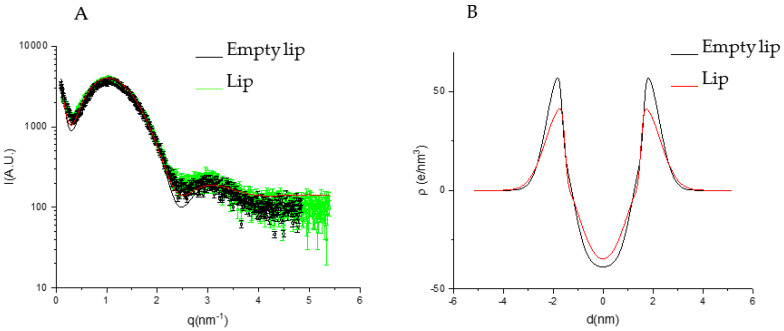
(**A**) SAXS profiles of empty and *P. spinosa* loaded liposomes. The lines correspond to the best fit of Gaussian bilayer models. (**B**) Electron density profiles corresponding to the best fits of empty and *P. spinosa* loaded liposomes.

**Figure 4:**
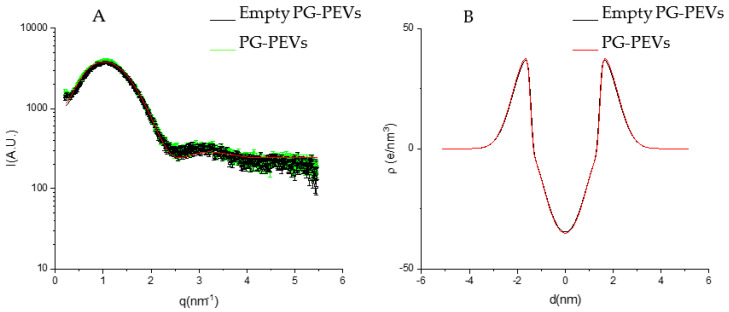
(**A**) SAXS profiles of empty and *P. spinosa* loaded PG-PEVs. The lines correspond to the best fit of Gaussian bilayer models. (**B**) Electron density profiles corresponding to the best fits of empty and *P. spinosa* loaded PG-PEVs.

A deeper structural characterization was performed by means of SAXS analyses. The SAXS profiles of liposomes and PG-PEVs (Figure 3 and Figure 4), together with the fits of the lamellar model, showed electronic density profiles typical of bilayers, as described in the literature [24,25,27,28]. The main parameters derived from the fits are reported in Table 7.

According to the model, all the vesicles were unilamellar (*N* = 1).

*Z_H_*, that is the distance between the polar heads and the bilayer center, slightly increased upon the extract’s loading, especially in liposomes, while the presence of PG reduced *Z_H_* values compared with liposomes. This is congruent with a main localization of the extract in the apolar part of the liposome bilayer, while PG in PEVs increases the hydrophilic part, which translates to an increase of area per molecule concomitant with the reduction in hydrophobic length.

The polar head region dimension, expressed by *σ_H_*, slightly increased in liposomes with the extract’s loading, indicating some participation of the extract in this region. The PG in PEVs increased the *σ_H_* value compared with liposomes, also congruent with a localization of PG in this region due to its miscibility with water. On the other hand, there were no differences between empty and extract-loaded PG-PEVs.

*σ_C_*, related to the segregation of terminal methyl groups at the center of the bilayer, had widely different values (Table 7). However, its effect on the hydrophobic electron profile was similar, that is, small or large values of *σ_C_* both corresponded to the absence of terminal methyl segregation in the bilayer (Figure 3A and Figure 4A).

### 3.5. Biocompatibility Evaluation

The biocompatibility of *P. spinosa* fruit extract was firstly determined via the hemolytic activity evaluation in erythrocytes.

The extract solution was tested from 200 to 2000 µg/mL and no relevant hemolytic activity was determined. For this reason, the two higher concentrations (1000 and 2000 μg/mL) were assayed for the nanoformulations.

All samples showed low erythrocyte-disrupting ability. Indeed, the hemolytic activity was lower than 3%, as reported in Table 8. At the concentration of 1000 μg/mL, the hemolytic activity was ≤2%, with non-statistically significant differences between the solution and the nanoformulations. At the concentration of 2000 μg/mL, the hemolytic activity of the extract solution increased, approximately 2.6%; this value was lowered to 1.2% by the extract-loaded liposomes (not statistically significant) and to 0.7% by the extract-loaded PG-PEVs (with statistical significance).

**Figure 5:**
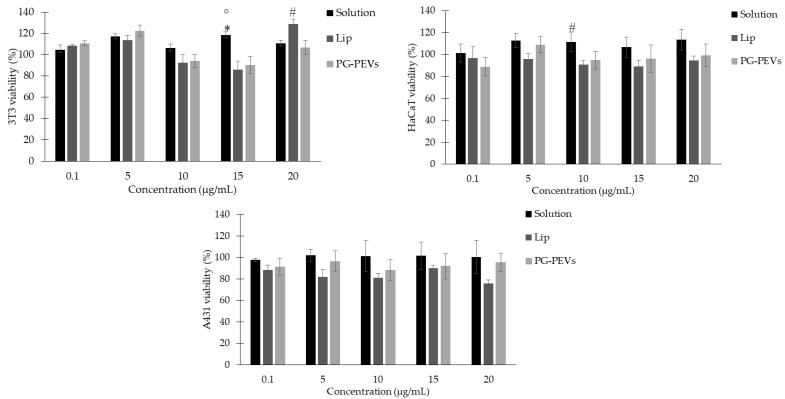
Viability of 3T3, HaCaT, and A431 cells upon exposure to *P. spinosa* samples for 24 h. Data are expressed as means ± standard error (SE) from two independent experiments, each performed in triplicate. For 3T3: * *p* < 0.05 vs. lip, ° *p* < 0.05 vs. PG-PEVs, # *p* < 0.05 vs. PG-PEVs; for HaCaT: # *p* < 0.05 vs. lip.

Further insights into the biological characterization of the nanoformulations included their interaction with skin cells. Among the putative cell lines suitable for the present study, commercially available cell lines that include representative cells of the different layers of the skin were chosen, such as fibroblasts (3T3) and keratinocytes with either non-tumoral (HaCaT) and tumoral (A431) characteristics. The absence of cytotoxic effects of the nanoformulations was assessed via the MTT test. The extract in the free form (solution) had no toxicity at all in the concentrations and in all the cell lines tested (Figure 5).

Considering 3T3 fibroblasts, the cell viability values were similar between cells treated with low concentrations (1–100 µg/mL) of the extract solution and the nanoformulations (liposomes or PG-PEVs). By increasing the concentration, some statistically relevant differences between the free extract and the liposomes or PG-PEVs at 150 µg/mL, and between the liposomes and the PG-PEVs at 200 µg/mL were observed (Figure 5).

When normal keratinocytes were considered (HaCaT), no relevant differences among the three samples were detected. Cell viability values ranged between 89 and 114%, and no significant differences against control cells were obtained (Figure 5).

Considering the tumoral keratinocytes (A431), a similar trend to normal keratinocytes was observed (Figure 5). In this case, however, the cell viability values upon exposure to liposomes were slightly lower, ranging between 76 and 90%, though not statistically different from the values obtained upon exposure to the solution and the PG-PEVs.

## 4. Discussion

The phytochemical characterization of *P. spinosa* fruit extract allowed us to identify phenolic compounds, also found in previous studies, with biological importance, as confirmed by the evaluation of the antioxidant and antibacterial activities. The extract showed antioxidant activity at a lower concentration than that required to exert an antibacterial activity (50 µg/mL and 16 mg/mL, respectively) under the experimental conditions tested. Our results are aligned with previous findings. The antioxidant activity of *P. spinosa* fruit extract was investigated in previous studies as a function of the scavenging of DPPH radicals. Coppari et al. showed that an ethanol extract acted as an antioxidant in a dose-dependent manner with an EC50 value of 64.2 µg/mL [6]. Considering the reported experimental conditions, the extract solution at 50 µg/mL inhibited approximately 1.7 × 10^−8^ moles of the DPPH solution (43% inhibition) [6]. Similar results were found by Sabatini et al.: an ethanol berry extract at 50 µg/mL could inhibit approximately 1.2 × 10^−8^ moles of the DPPH solution (approximately 30% inhibition) [40]. Our extract solution at 50 µg/mL inhibited 4.2 × 10^−8^ moles of the DPPH solution (85% inhibition), therefore an amount 2.5- and 3.5-fold higher, respectively.

Sabatini et al. also described the antibacterial activity of a *P. spinosa* fruit ethanol extract against *S. aureus*, finding an MBC value (17.44 mg/mL) similar to the value reported in this study (16 mg/mL). Moreover, the Authors found a wider antimicrobial activity for this extract, defining it as a promising antimicrobial compound of natural origin [40].

Of note, no previous data were found on the inhibitory activity of *P. spinosa* fruit extract against *S. epidermidis*.

As widely reported in the literature, the biological potential of plant compounds is often limited by several issues that could be overcome by the incorporation in delivery systems. Particularly, a previous study [11] demonstrated how the incorporation of *P. spinosa* fruit extract in biomimetic nanoparticles could improve its anti-inflammatory properties.

In the present study, two types of phospholipid vesicles were formulated for the prepared extract, to enhance local bioavailability and facilitate application on the skin [16]. Both formulations had an extract concentration of 20 mg/mL and a size below 100 nm, with PG leading to the formation of vesicles smaller than liposomes (86 vs. 94 nm). Both vesicles had similar polydispersity index and zeta potential values, and unilamellar structure, confirmed by electron microscopy and SAXS results. Compared to the *P. spinosa* biomimetic nanoparticles [11], our liposomes and PG-PEVs showed a smaller size (140 and 125 nm for PSF-DOPCs and PSF-DOPGs vs. 94 and 86 nm for our liposomes and PG-PEVs) and a higher entrapment efficiency for the same compounds identified (1–56% for PSF-DOPCs and PSF-DOPGs vs. 24–99% for liposomes and PG-PEVs). Therefore, it can be concluded that the lipid composition of the nanoformulations critically influences size and entrapment efficiencies. In our study, the entrapment efficiency of the main compounds present in the extract was high, especially for anthocyanins and flavonols (71–99%). Although highly represented in the extract, hydroxycinnamic acids were entrapped into vesicles with less efficiency (24–38%). This selectivity was maintained for both liposomes and PG-PEVs, with the latter showing lower entrapment efficiencies than the former, with a decrease in efficiency between 7 and 20%. Therefore, the addition of PG to the formulation led to a reduction of the vesicles’ size and of the entrapment efficiency. These differences could be explained by the different hydrophilic/polar characters of the marker phenolic compounds and by electronic interactions between the phenolic compounds and the utilized phospholipids [11], thus providing hints for the nanoformulation selection.

The electron scattering profile obtained with X-ray diffraction suggests that the extract and the penetration enhancer PG did not largely affect the bilayer structures, as highlighted by similar values for the parameters analyzed. Given the high entrapment efficiencies, two possible explanations are:

- The extract is mainly localized in the hydrophilic core of the vesicles;
- The extract does not affect the bilayer structure because of the low concentration or the low electron density contrast.

The biocompatibility of the proposed nanoformulations was tested as the hemolytic response on erythrocytes, one of the most widely used cell membrane systems [41], providing information about the safety of new systems kept in contact with blood. The extract had no hemolytic activity, since the values were below 5% [42,43], and even lower when it was nanoformulated. Some results found in the literature, although regarding aqueous extract obtained from *P. spinosa* berries, highlighted a hemolysis inhibition in human erythrocytes under oxidative stress conditions [4], and an inhibition capacity of the oxidative haemolysis of erythrocytes from healthy sheep [5].

The biocompatibility of the *P. spinosa* fruit extract nanoformulations was further demonstrated in skin model cells. Fibroblasts (3T3) have become one of the most common non-epithelial cell lines used in in vitro studies on general cytotoxicity and biocompatibility studies. Swiss albino mouse fibroblasts are readily available, show a well-defined protocol, and represent a physiological model cell line. Keratinocytes represent 95% of the epidermal cells, acting as structural and barrier functions of the epidermis. The spontaneously immortalized human keratinocytes HaCaT cell line from adult skin has been proposed as a model for the study of keratinocytes functions. This work also includes keratinocytes with tumoral characteristics, such as squamous cell carcinoma (SCC) through the A431 cell line, considering that SCC is by far the most common skin cancer and is more common than any other form of cancer. The biocompatibility was proved by the absence of cytotoxicity in the three cell lines, thus supporting the potential biomedical application of the *P. spinosa* nanoformulations.

## 5. Conclusions

The nanoformulation of natural extracts is an effective strategy to overcome issues related to undesirable features of bioactive compounds. We formulated a *P. spinosa* fruit extract in phospholipid vesicles by employing a simple method that involves the sonication of the phospholipid and the extract in a dispersing medium. Importantly, we obtained vesicles with a high entrapment efficiency of phenolic compounds, characteristic of the extract. Moreover, the vesicles were small in diameter, and this is an important matter because the particle size is a key factor for the transport of the active compounds through the skin layers, having an impact on their stability, release, and cellular uptake.

The biological properties (i.e., antioxidant and antibacterial) of the extract were probed, and the biocompatibility of the nanoformulations was demonstrated, which points to the promising perspective of confidently using them for further investigations on biological efficacy.

## Figures and Tables

**Table 1:** Composition of the *P. spinosa* fruit extract nanoformulations.

	Lecithin	Extract	PG	Water
Lip	180 mg	20 mg		1 mL
Empty lip	180 mg			1 mL
PG-PEVs	180 mg	20 mg	100 µL	900 µL
Empty PG-PEVs	180 mg		100 µL	900 µL

**Table 2:** Compounds identification by (HR) LC-ESI-QTOF MS/MS in *P. spinosa* fruit extract.

Compound No.	Rt min	Identity	[M-H]^− §^ *m*/*z*	Molecular Formula	Δ ppm	MS/MS ^§,^* m/*z*	References	Identification Confidencelevel ^#^
1	6.59	vanillic acid-*O*-glucopyranoside	329.0877	C_14_H_18_O_9_	−0.2134	167.0349(100)	[1,32]	2
2	6.83	caffeoylquinic acid	353.0879	C_16_H_18_O_9_	−0.0047	191.0561(78)/179.0342(44)	[1]	2
3	7.43	phenolic glycoside	313.0932	C_14_H_18_O_8_	-	59.0140(100)/101.0245(39)	[18]	3
4	7.68	3-*O*-caffeoylquinic acid	353.0885	C_16_H_18_O_9_	−0.3257	191.0563(100)/179.0353(53)	[1,32,33,34]	1
5	9.21	coumaroylquinic acid isomer	337.0930	C_16_H_18_O_8_	−0.3320	163.0401(100)/119.0505(64)	[18]	2
6	9.74	3-*p*-coumaroylquinic acid	337.0940	C_16_H_18_O_8_	−0.2511	163.0403(100)/119.0504(48)	[1,32,33,34]	2
7	10.27	unknown	469.0979	C_20_H_22_O_13_	-	191.0558(100)/59.0140(11)	-	4
8	10.53	caffeic acid hexoside	341.0878	C_15_H_18_O_9_	0.0021	179.0346(100)/135.0448(35)	[1]	2
9	10.67	unknown	391.1251	C_16_H_24_O_11_	-	44.9995(100)	-	4
10	10.84	caffeoylquinic acid hexoside	515.1399	C_22_H_28_O_14_	−1.370	179.0339(100)/341.0853(35)/191.0554(17)	[18]	3
11	11.18	3-*O*-feruloylquinic acid	367.1043	C_17_H_20_O_9_	−0.4858	193.0516(100)/134.0372(77)	[1,32,33,34]	2
12	11.33	5-*O*-caffeoylquinic acid	353.0884	C_16_H_18_O_9_	0.5943	191.0561(100)/179.0347(55)	[1,32,34]	1
13	11.55	hydroxybenzoyl-hexosyl-hexoside	461.1302	C_19_H_26_O_13_	−0.4844	121.0295(100)	[32,35]	3
14	11.73	barlerin isomer I	447.1508	C_19_H_28_O_12_	−0.7799	71.0141(100)/101.0242(41)	[32,36]	3
15	12.29	barlerin isomer II	447.1508	C_19_H_28_O_12_	−0.7799	101.0242(100)/71.0146(46)	[32,36]	3
16	13.62	4-*p*-coumaroylquinic acid	337.0940	C_16_H_18_O_8_	−0.2511	173.0461(100)/163.0403(32)	[1,32,33,34]	2
17	14.94	caffeoylshikimic acid isomer I	335.0773	C_16_H_16_O_8_	0.0590	179.0352(100)/135.0448(75)/161.0247(43)	[1,32,37]	2
18	15.59	caffeoylshikimic acid isomer II	335.0773	C_16_H_16_O_8_	0.1590	161.0247(100)/135.0448(20)/179.0339(15)	[1,32,37]	2
19	16.15	ethyl caffeoylquinate	381.1186	C_18_H_22_O_9_	−0.5058	161.0246(100)	[32,38]	3
20	18.89	quercetin hexosyl-rhamnoside	609.1468	C_27_H_30_O_16_	0.6916	300.0272(100)/301.0344(39)/343.0440(21)	[1,32,34]	2
21	19.25	quercetin-3-*O*-rutinoside	609.1458	C_27_H_30_O_16_	−0.3084	300.0269(100)/301.0342(60)/343.0458(17)	[1,32,33,34]	1
22	19.48	quercetin-3-*O*-glucoside	463.0886	C_21_H_20_O_12_	0.4004	300.0270(100)/301.0322(41)	[1,32,33,34]	1
23	19.70	quercetin pentosyl-hexoside	595.1303	C_26_H_28_O_16_	−0.1584	300.0280 (100)/301.0322(15)/415.0632(12)	[1,32,33,34]	2
24	19.83	quercetin hexoside	463.0879	C_21_H_20_O_12_	−0.2996	300.0260(100)/301.0363(37)	[32,34]	2
25	20.73	quercetin pentoside	433.0773	C_20_H_18_O_11_	−0.3349	300.0277(100)/301.0366(37)	[1,32,34]	2
26	21.18	quercetin pentoside	433.0779	C_20_H_18_O_11_	−0.3249	300.0280(100)	[1,32,33]	2
27	21.54	quercetin pentoside	433.0784	C_20_H_18_O_11_	0.7651	300.0274(100)/301.0354(72)	[1,32,33]	2
28	21.68	quercetin hexosyl-rhamnoside	609.1457	C_27_H_30_O_16_	−0.4084	301.0360(100)/300.0255(98)	[1,32,33]	2
29	22.09	quercetin-3-*O*-rhamnoside	447.1006	C_21_H_20_O_11_	0.0427	300.0267(100)/301.0351(71)	[1,32,33,34]	1
30	22.63	quercetin acetyl hexoside	505.0992	C_23_H_22_O_13_	0.3318	300.0261(100)/271.0227(30)	[1,32,34]	2
31	25.96	quercetin acetyl hexosyl-rhamnoside	651.1561	C_29_H_32_O_17_	−0.5137	301.0349(100)/300.0267(50)/609.1430(15)	[1,32,34]	2

^§^ experimental results; * in brackets the relative intensity; ^#^ according to Blaženović et al. [31].

**Table 3:** Concentration of targeted phenolic compounds detected in *P. spinosa* fruit extract (mg/g of dried extract (dr), mean ± SD; *n* = 3).

Compound	Peak No. ^§^	*P. spinosa* Extract (mg/g dr)	
		Mean	±SD
**Total Anthocyanins**		**1.72**	**0.05**
cyanidin-3-*O*-glucoside	A1	0.43	0.01
cyanidin-3-*O*-rutinoside	A2	0.74	0.03
peonidin-3-*O*-glucoside	A3	0.11	0.00
peonidin-3-*O*-rutinoside	A4	0.44	0.01
**Total Flavonols**		**1.33**	**0.01**
quercetin-3-*O*-rutinoside	21	0.31	0.00
quercetin-3-*O*-glucoside	22	0.06	0.00
quercetin-3-*O*-rhamnoside	29	0.12	0.00
quercetin pentosides ^a^	(25, 26, 27)	0.49	0.00
Other quercetin derivatives ^a^	(20, 23, 24, 28, 30, 31)	0.35	0.00
**Total Hydroxycinnamic acids**		**2.72**	**0.02**
3-*O*-caffeoylquinic acid	4	2.38	0.02
3-*p*-coumaroylquinic acid ^b^	6	0.13	0.00
3-*O*-feruoilquinic acid ^c^	11	0.07	0.00
5-*O*-caffeoylquinic acid	12	0.13	0.00
**Total Hydroxybenzoic acids**		**0.12**	**0.00**
vanillic acid-*O*-glucopyranoside ^d^	1	0.12	0.00
**Total polyphenols**		**5.92**	**0.08**

^a^ expressed as quercetin-3-*O*-glucoside equivalents; ^b^ expressed as *p*-coumaric acid equivalents; ^c^ expressed as ferulic acid equivalents; ^d^ expressed as vanillic acid equivalents; ^§^ peak number as reported in Table 2 (1–31) and Figure S1 (A1–A4).

**Table 4:** Minimum Inhibitory Concentration (MIC) and Minimum Bactericidal Concentration (MBC) of *P. spinosa* fruit extract.

Bacteria Strains	MIC (mg/mL)	MBC (mg/mL)
*Bacillus subtilis*	/	/
*Staphylococcus epidermidis*	16	16
*Staphylococcus aureus*	16	16
*Listeria monocytogenes*	/	/
*Enterococcus faecalis*	/	/
*Escherichia coli*	/	/
*Acinetobacter baumannii*	/	/
*Klebsiella aerogenes*	/	/

**Table 5:** Characteristics of *P. spinosa* fruit extract nanoformulations. Each value represents the mean ± standard deviation (SD); *n* > 10.

	Lip	Empty Lip	PG-PEVs	Empty PG-PEVs
Mean diameter (nm ± SD)	** 94 ± 2.0	70 ± 2.6	**^,#^ 86 ± 6.4	59 ± 3.3
Polydispersity index (± SD)	0.19 ± 0.01	0.19 ± 0.03	0.21 ± 0.02	0.21 ± 0.03
Zeta potential (mV ± SD)	−48 ± 1.4	−46 ± 2.3	−47 ± 1.7	−47 ± 1.1

** Values statistically different (*p* < 0.01) from the corresponding empty vesicles. ^#^ Values statistically different (*p* < 0.05) from liposomes.

**Table 6:** Entrapment efficiency (EE %) of *P. spinosa* fruit extract nanoformulations. Data are given as means ± standard deviations (SD); *n* = 4.

Peak No. ^§^	Compound	EE% ± SD	EE% ± SD
		Lip	PG-PEVs
2	3-*O*-caffeoylquinic acid	33.6 ± 3.4	24.2 ± 1.4
4	3-*O*-feruoilquinic acid ^a^	35.3 ± 4.3	28.2 ± 1.7
5	5-*O*-caffeoylquinic acid	38.0 ± 3.4	28.2 ± 1.5
A1	cyanidin-3-*O*-glucoside	98.6 ± 0.9	84.9 ± 1.7
A2	cyanidin-3-*O*-rutinoside	96.3 ± 0.9	74.4 ± 1.7
A4	peonidin-3-*O*-rutinoside	97.0 ± 0.5	77.4 ± 0.9
14	quercetin-3-*O*-rutinoside	82.6 ± 6.1	64.5 ± 3.2
15	quercetin-3-*O*-glucoside	85.1 ± 7.8	72.8 ± 1.7
20	quercetin pentoside ^b^	87.5 ± 7.5	70.7 ± 4.8

^a^ expressed as ferulic acid equivalents; ^b^ expressed as quercetin-3-*O*-glucoside equivalents. ^§^ peak number as reported in Table 2.

**Table 7:** Fitting parameters and derived parameters (±estimated error from the fit) for SAXS curves of empty and *P. spinosa* nanoformulations.

	Lip	Empty Lip	PG-PEVs	Empty PG-PEVs
*χ* ^2^	6.36	5.07	3.42	3.72
*Z_H_* (Å)	18.40 ± 0.5	17.27 ± 0.5	15.49 ± 0.5	15.29 ± 0.5
*σ_H_* (Å)	5.91 ± 0.5	5.30 ± 0.5	6.43 ± 0.5	6.47 ± 0.5
*σ_C_* (Å)	17.73 ± 10	4.62 × 10^−5^ ± 1	4.27 × 10^−4^ ± 1	4.27 × 10^−4^ ± 1

**Table 8:** Hemolytic activity of *P. spinosa* fruit extract in solution and in the nanoformulations. Data are expressed as % means ± standard deviations (SD); *n* = 2.

	Extract Concentration (µg/mL)	Hemolytic Activity (% ± SD)
Solution	1000	2.00 ± 0.32
Lip	1000	0.66 ± 0.46
PEVs	1000	0.66 ± 0.52
Solution	2000	2.61 ± 0.71
Lip	2000	1.20 ± 0.62
PEVs	2000	* 0.65 ± 0.06

* *p* < 0.05 vs. extract solution 2000 µg/mL.

## Data Availability

The data presented in this study are available within this article.

## Supplementary Materials

The following supporting information can be downloaded at: https://www.mdpi.com/article/10.3390/pharmaceutics15041063/s1, Figure S1: LC-DAD chromatogram of *P. spinosa* extract at λ = 520 nm.
