# Supplementary material for: Phenolic Fingerprint, Bioactivity and Nanoformulation of Prunus spinosa L. Fruit Extract for Skin Delivery
Source: Pharmaceutics. 2023 Mar 25;15(4):1063. doi: 10.3390/pharmaceutics15041063 (PMC10144133; doi:10.3390/pharmaceutics15041063)
Supplement: Supplementary file 1 [file pharmaceutics-15-01063-s001.zip › pharmaceutics-2234246-supplementary.pdf]

pharmaceutics

MDPI

Supplementary Material

Phenolic Fingerprint, Bioactivity and Nanoformulation of *Prunus spinosa* L. Fruit Extract for Skin Delivery

Maria De Luca [1,2], Carlo Ignazio Giovanni Tuberoso [3], Ramon Pons [4], María Teresa García [4], Maria del Carmen Morán [5,6], Giulio Ferino [7], Antonio Vassallo [1,8], Giuseppe Martelli [1], Carla Caddeo [3,*]

1 Department of Science, University of Basilicata, Viale dell'Ateneo Lucano 10, 85100 Potenza, Italy
2 KAMABIO Srl, Via Al Boschetto 4/B, 39100 Bolzano, Italy
3 Department of Life and Environmental Sciences, University of Cagliari, SS 554 – bivio per Sestu, Monserrato, 09042 Cagliari, Italy
4 Department of Surfactants and Nanobiotechnology, Institute for Advanced Chemistry of Catalonia (IQAC-CSIC), c/Jordi Girona, 18-26, 08034 Barcelona, Spain
5 Department of Biochemistry and Physiology, Physiology section, Faculty of Pharmacy and Food Science, University of Barcelona, Avda. Joan XXIII 27-31, 08028 Barcelona, Spain
6 Institute of Nanoscience and Nanotechnology - IN2UB, University of Barcelona, Avda. Diagonal, 645, 08028 Barcelona, Spain
7 CeSAR, University of Cagliari, SS 554 – bivio per Sestu, Monserrato, 09042 Cagliari, Italy
8 Spinoff TNcKILLERS s.r.l., Viale dell'Ateneo Lucano 10, 85100 Potenza, Italy
* Correspondence: caddeoc@unica.it

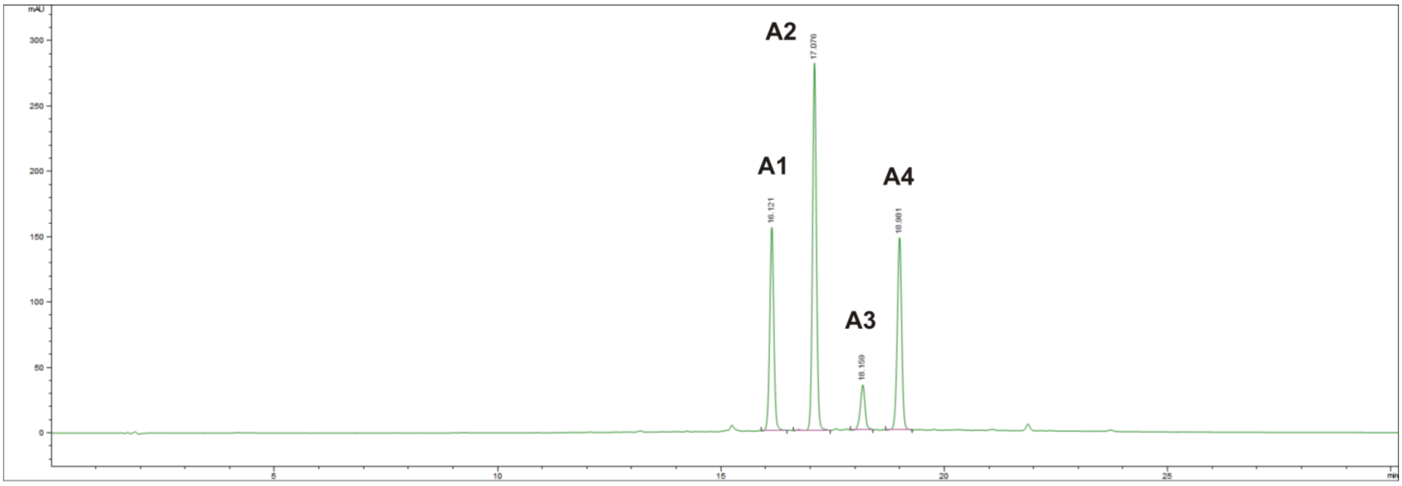

Figure S1. LC-DAD chromatogram of *P. spinosa* extract at λ = 520 nm. Peaks identification: A1: cyanidin-3-*O*-glucoside, A2: cyanidin-3-*O*-rutinoside, A3: peonidin-3-*O*-glucoside, and A4: peonidin-3-*O*-rutinoside. Chromatographic conditions are reported in the main text.
